# Conformational Change in the Chromatin Remodelling Protein MENT

**DOI:** 10.1371/journal.pone.0004727

**Published:** 2009-03-06

**Authors:** Poh Chee Ong, Sarah J. Golding, Mary C. Pearce, James A. Irving, Sergei A. Grigoryev, Debbie Pike, Christopher G. Langendorf, Tanya A. Bashtannyk-Puhalovich, Stephen P. Bottomley, James C. Whisstock, Robert N. Pike, Sheena McGowan

**Affiliations:** 1 Department of Biochemistry and Molecular Biology, Monash University, Clayton, Victoria, Australia; 2 Department of Biochemistry and Molecular Biology, Penn State University College of Medicine, Milton S. Hershey Medical Center, Hershey, Pennsylvania, United States of America; Temasek Life Sciences Laboratory, Singapore

## Abstract

Chromatin condensation to heterochromatin is a mechanism essential for widespread suppression of gene transcription, and the means by which a chromatin-associated protein, MENT, induces a terminally differentiated state in cells. MENT, a protease inhibitor of the serpin superfamily, is able to undergo conformational change in order to effect enzyme inhibition. Here, we sought to investigate whether conformational change in MENT is ‘fine-tuned’ in the presence of a bound ligand in an analogous manner to other serpins, such as antithrombin where such movements are reflected by a change in intrinsic tryptophan fluorescence. Using this technique, MENT was found to undergo structural shifts in the presence of DNA packaged into nucleosomes, but not naked DNA. The contribution of the four Trp residues of MENT to the fluorescence change was mapped using deconvolution analysis of variants containing single Trp to Phe mutations. The analysis indicated that the overall emission spectra is dominated by a helix-H tryptophan, but this residue did not dominate the conformational change in the presence of chromatin, suggesting that other Trp residues contained in the A-sheet and RCL regions contribute to the conformational change. Mutagenesis revealed that the conformational change requires the presence of the DNA-binding ‘M-loop’ and D-helix of MENT, but is independent of the protease specificity determining ‘reactive centre loop’. The D-helix mutant of MENT, which is unable to condense chromatin, does not undergo a conformational change, despite being able to bind chromatin, indicating that the conformational change may contribute to chromatin condensation by the serpin.

## Introduction

The **M**yeloid and **E**rythroid **N**uclear **T**ermination stage specific protein, MENT, is a member of the serpin superfamily [1] and is capable of inhibiting the papain-like cysteine proteases, cathepsins V and L [2]–[4]. Inhibitory serpins are metastable molecules that utilise a conformational rearrangement to inhibit target enzymes: upon interaction with a protease, the solvent-exposed reactive centre loop (RCL) is cleaved, but remains covalently bound to the protease as an acyl enzyme intermediate as it inserts into the centre of the large central β-sheet (the A-sheet) to form an additional β-strand. In the final serpin-enzyme complex, the protease is trapped in a distorted, inactive conformation [5].

MENT is also a potent chromatin remodelling protein that is responsible for heterochromatin spreading and control of terminal cell differentiation in avian erythrocytes [6]. The regions of MENT primarily responsible for interaction with nucleosomal DNA is an interhelical extension termed the M-loop, and an area centred on the D- and E-helices [3]. The genetic material of eukaryotic cells is packaged in the form of chromatin: a nuclear mass composed of DNA, histones and other associated protein. Controlling the level of compaction of chromatin is essential for the regulation of all cellular processes.

Several lines of evidence suggest that there may be a relationship between the inhibitory activity of MENT and its role in condensing chromatin: (1) protease inhibition by MENT contributes to chromatin rearrangements *in vivo* [2], [7]; (2) cathepsin V, a nuclear protease implicated in the control of the transcription factor CDP/CUX, is rendered susceptible to inhibition by MENT in the presence of DNA [8]; and (3) MENT is able to form protein-protein bridges, mediated by the RCL, that may be important for chromatin remodelling [3].

In the crystal structure of wild-type MENT, two residues of the RCL are inserted into the top of the A β-sheet. Most serpins characterised to date that exhibit such partial RCL insertion undergo co-factor mediated conformational shifts; for example antithrombin and heparin co-factor II both undergo RCL expulsion in the presence of heparin. Therefore, we have used DNA, both naked and nucleosome-bound, to investigate conformational change in MENT by measuring changes in intrinsic tryptophan fluorescence. It was found that soluble chromatin was able to induce conformational change in MENT in contrast to naked DNA that was unable to induce any change. To attempt to determine the basic structure required for a conformational change in MENT, we constructed mononucleosomes and found that this was the necessary structure required for a conformational change in the molecule. We further probed this conformational rearrangement by analysing the changes seen in key MENT binding mutants and deconvolution of the tryptophan emission spectra using single tryptophan variants of MENT.

## Results and Discussion

### MENT_WT_ undergoes a conformational change in the presence of chromatin that is independent of the RCL sequence but dependent upon the M-loop and D-helix residues

The mechanism by which nucleosome arrays are folded into higher order structures remains somewhat obscure. While the structures of individual nucleosomes and chromatin remodelling proteins have proved essential to understanding chromatin structure, traditional crystallographic approaches to higher order chromatin structure determination have been hampered by a lack of homogeneity as well as mobility in higher order chromatin structures. Thus, biophysical studies have proved central to our understanding of protein mobility during chromatin remodelling [9], [10].

Conformational flexibility in serpins is well characterised and represents a crucial evolutionary advantage of their function [11]. In order to ascertain if the interaction of MENT with chromatin during condensation induced any structural changes within the serpin, we analysed the intrinsic tryptophan fluorescence of MENT and key variants in the presence and absence of purified soluble chromatin (Table 1), which is fluorescently silent in the tryptophan range (data not shown). An analogous approach has successfully been used to measure conformational change in the presence of heparin for antithrombin [12].

**Table 1:** Change in fluorescence intensity of MENT and mutant proteins.

Protein	Alteration location	Chromatin (S2)^a^	Positioning DNA^a^	Mononucleosomes^a^
Wildtype		+25.6±3.4	NC	+19.3±4.0
MENT_OV_	RCL	+22.8±2.4	NC	+13.1±3.3
MENT_ΔMloop_	M-loop	NC	NC	−13.6±2.8
MENT_M3_	D-helix	NC	NC	−21.4±7.8

a Percentage change (%) in fluorescence intensity calculated at 340 nm. All experiments are the average of three independent replicates. Abbreviation (NC) = no change.

The emission spectra of tryptophan residues reports changes in their surrounding environment. Excitation of the tryptophan residues in MENT_WT_ at 295 nm revealed a significant increase in fluorescence intensity at a peak of 340 nm (typical of the tryptophan indole ring fluorescence) upon addition of chromatin (Table 1). In contrast, the deleted M-loop variant (MENT_ΔMloop_), that is unable to properly interact with DNA or chromatin, showed no change in tryptophan fluorescence in the presence of chromatin (Table 1). MENT_OV_, a mutant in which the RCL had been replaced with that of the non-inhibitory serpin, ovalbumin, showed a comparable increase in fluorescence to the wild-type protein (Table 1). These data indicate that the RCL sequence is not essential for the chromatin-induced conformational change in MENT, but the presence of the M-loop is. This was not totally unexpected as the M-loop is essential for DNA binding. However, the final key variant of MENT that we tested is a mutant that carries a triple mutation within the D-helix of the protein (MENT_K99Q,R107Q,K109Q_ or MENT_M3_). We have previously shown that MENT variants carrying this alteration are capable of binding DNA/chromatin, but lack the ability to properly condense into nucleoprotein complexes as the wild-type protein can [3]. Interestingly, although able to bind to the cofactors tested, the MENT_M3_ mutant was unable to undergo any conformational change in the presence of chromatin and exhibited a spectra that was the same as the non-DNA-binding profile.

### Conformational change in MENT requires DNA to be packaged in nucleosomes

We have previously shown that naked DNA does not cause a conformational change in MENT [3]. We repeated these experiments using the 207 bp mononucleosome positioning DNA fragment [13]. These data confirmed that wild-type protein, MENT_ΔMloop_, MENT_OV_ and MENT_M3_ did not undergo a conformational change in the presence of mononucleosome positioning DNA alone (Table 1), despite the fact that all of the serpin variants except MENT_ΔMloop_ were capable of binding the cofactor. We attempted to analyse the effect of the nucleosomal core histones on the fluorescence of MENT by the addition of soluble core histones isolated from chicken nuclei. The results indicated that there was no change in fluorescence (data not shown), however in the absence of DNA, histones form stable octamers only at 2 M salt concentration, hence under assay conditions, these results are indicative of a mixed population of octamer components and may not be conclusive.

Thus it appeared that the two major components of chromatin, DNA and histone proteins, were not capable of inducing a conformational change in MENT, in contrast to soluble chromatin. Previous studies have shown that the wild-type MENT protein shows a high affinity for binding to the DNA linker stem structure of mononucleosomes [3], thus we tested the ability of reconstituted mononuclesomes to induce a conformational change in MENT. The intrinsic tryptophan emission spectra for wild-type MENT indicated that there was a modest increase in fluorescence at 340 nm in the presence of mononucleosomes (Table 1). Interestingly, the MENT_OV_ variant showed an equivalent conformational change to the wild-type protein, but the DNA binding mutant, MENT_ΔMloop_ and the condensation mutant, MENT_(M3)_, decreased (Table 1). It is likely that as the MENT_ΔMloop_ is unable to bind to the mononucleosome [3], the presence of the nucleoprotein material has lead to a shielding of the tryptophan fluorescence emission. The MENT_M3_ protein is able to bind to mononucleosomes, but does not induce the same nucleoprotein complex formation as the wild-type protein [3]. The spectral results for this mutant exhibited great variation in the results obtained (reflected by the significantly larger standard deviations of the replicates) and we presume that, like MENT_ΔMloop_, the effect noted is due to shielding of the tryptophans as a result of inappropriate nucleoprotein complex formation or a reduction in the formation of these complexes.

### Conformational flexibility of MENT

**Figure 1:**
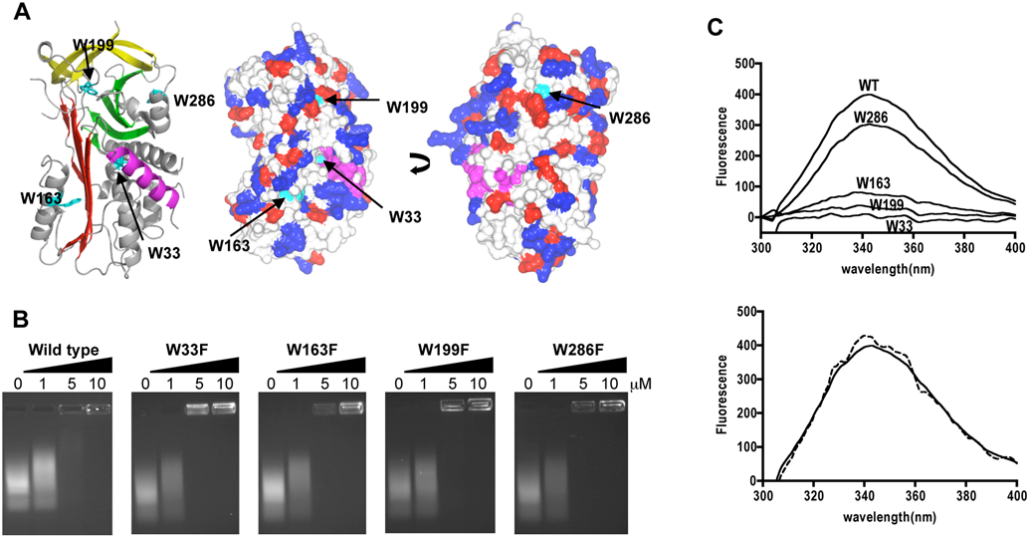
Deconvolution of the tryptophan emission spectra of MENT. (A) Structure of native wild type MENT showing the location of the four tryptophan residues. Cartoon of the location of the four tryptophan residues in the structure of native wild type MENT (2H4R.pdb; McGowan et al. 2006) also showing the A β-sheet (red), B β-sheet (green) and C β-sheet (yellow), D-helix (magenta) and helices hA-hI (grey). The electrostatic potential surface of native wild type MENT (CC4MG, [22], [23]) with tryptophan residues shown in cyan and the D-helix in magenta. (B) Chromatin association assays with increasing concentrations of wild type MENT and tryptophan variants. The final concentration (µM) of purified protein, as indicated at the top of each panel, added to soluble erthrocyte chromatin (OD_260_ = 1.6). (C) Deconvolution of the fluorescence emission of wild type MENT. The deconvolution of the spectrum of MENT (0.2 µM) into contributions of individual tryptophan residues. The independent behaviour of tryptophan residues in MENT where the *solid line* is the spectrum of the wild-type MENT (0.2 µM), and the *dashed line* represents one-third of the sum of the spectra of individual tryptophan variants.

The positions of the tryptophan residues in the native wild-type MENT structure shows that two residues are located in the shutter region (W33 on the B-helix and W163 of the F-helix), one is located in the breach (W199, at the end of strand s3A) and W286 is located on the H-helix (Fig 1A). The indole ring of W163 is significantly more solvent-exposed than those of W286, W33 or W199 (Fig 1A).

We replaced the tryptophan residues with phenylalanine to construct four MENT individual variants (W33F, W163F, W199F and W286F). All four variants were found to be able to condense chromatin with the same affinity as the wild-type protein (Fig 1B). The integrity of the MENT mutants was verified using: (1) far-UV circular dichroism spectroscopy (data not shown); (2) thermal stability; and (3) inhibitory activity (the association rate constant, *k*_ass_, and the stoichiometry of inhibition, SI) against human cathepsin V (Table 2). While W163F was shown to have a lower thermal stability than the other proteins, with its thermal stability reduced by a maximum of 5°C, crucially, all mutant proteins retained both inhibitory activity and chromatin condensing activity. As had been determined previously for other proteins [14], [15], multiple tryptophan to phenylalanine replacements caused a serious reduction in stability of the variants (data not shown) and therefore variants containing multiple mutations were not analysed further in this study.

**Table 2:** Characterisation and fluorescence intensity of tryptophan mutant proteins.

Protein	Alteration location	SI	*k*_ass_ a	T_m_ b	Change in fluorescence intensity^c^ (%) in presence of Chromatin (S2)
MENT_(W33F)_	hB	1.4	2.0±0.15	68	U
MENT_(W163F)_	hF	1.4	2.6±0.07	63	U
MENT_(W199F)_	s3A	1.3	3.7±0.11	65	U
MENT_(W286F)_	hH	3.0	1.7±0.13	67	+ 8.5 ± 0.2

a (M^−1^s^−1^ (×10^5^).

b Melting temperature (T_m_ in °C) is defined as the inflection point of the thermal unfolding.

c Percentage (%) change in fluorescence intensity calculated at 340 nm. Abbreviations are as follow (NC) = no change; (∼) = not determined; (U) = uninterpretable. All experiments are the average of three independent replicates.

Steady state tryptophan emission spectra were recorded for each mutant upon excitation at 295 nm. Each variant gave an emission spectrum with lower fluorescence intensity than the wild-type protein. To determine the contribution of each of the tryptophan residues to the overall spectrum, we deconvoluted the spectrum obtained by subtracting each individual tryptophan spectrum from that of the wild type spectrum (Fig 1C). The results indicated that W286 was the predominant contributor (72%) to fluorescence in the wild type protein, followed by W163 (18%). The W199 and W33 residues only had minor contributions of 9% and 1.0%, respectively.

Accurate deconvolution of the emission spectra requires that each tryptophan contribute independently to the emission spectrum. To determine if each tryptophan contributed independently to the emission spectrum of the wild type MENT protein, a comparison was made between the sums of the emission spectrum of the four mutants to that of wild type MENT. If the tryptophans did fluoresce independently of each other, the wild type spectrum should be identical to one-third of the sum of the four spectra of the single mutants [12] (Fig 1C). This was indeed the case, confirming that each tryptophan residue in MENT fluoresces independently of the other three.

We measured the effect of chromatin on the spectrum of each tryptophan variant. Unfortunately, however, due to the dominance of W286 to the emission, deconvolution analyses of W33, W163 and W199 were not interpretable. The contribution of W286 to the conformational change of MENT in the presence of chromatin was calculated to be only 8.5% (Table 2), in comparison to the ∼25% change observed with the wild-type protein. This indicates that the conformational change seen in MENT is not solely located in and around the H-helix and that the other three tryptophans located in the flexible breach and shutter regions must contribute to this change as well. Due to the dominant contribution of W286 to the overall spectra, further detailed mapping of the conformational change was not possible.

The inability of a D-helix (MENT_M3_) mutant to undergo any detectable conformational change suggests that it is possible that chromatin promotes RCL expulsion, in a similar fashion to the effect of heparin on antithrombin [16]. Binding of the correct cofactor (the nucleosome rather than DNA) may allow the molecule to move, releasing the RCL from constraints, shifting the body of the entire molecule as the loop is expelled [16], [17]. However, in the event of RCL expulsion, we might expect the kinetics of MENT interaction with target proteases to be substantially altered in the presence of chromatin, as seen with antithrombin and Factor Xa for example [18]. Interestingly, we have recently shown that the rate of inhibition of cathepsin V by MENT is significantly accelerated in the presence of DNA and also soluble chromatin [8]; however, our investigation into the mechanism of this rate enhancement indicated that this change in inhibition was actually controlled by a change in the protease rather than the inhibitor [8]. Thus, the precise structural effect of chromatin on MENT remains to be understood. This study has shown that nucleosomal DNA induces a conformational change in MENT that is dependent on correct binding to the M-loop and D-helix residues of the serpin. Although structural mapping studies were less informative than hoped, the results indicate that the A-sheet and RCL are likely to be involved. Interestingly, the D-helix mutant of MENT, which does bind DNA, but doesn't undergo conformational change, is unable to condense chromatin, suggesting that the conformational change induced in MENT may play a role in chromatin condensation carried out by this serpin.

## Materials and Methods

### Mutagenesis and analysis of MENT variants

Point mutations were introduced into the MENT_WT_ gene using the QuikChange approach (Novagen) using oligonucleotides (GeneWorks). MENT proteins were expressed and purified as described previously [3]. The concentration of the MENT mutants was confirmed through a Bradford assay (BioRad) in comparison to a MENT_WT_ standard curve [19]. Thermal unfolding and analysis was performed as previously described [20] using circular dichroism measurements on a Jasco 810 spectropolarimeter with a thermostatted cuvette at 25°C at a protein concentration of 0.02 mg/ml with a 0.1 cm path length. Far-UV CD spectra from 250 to 200 nm were collected with one second/point signal averaging.

### Chromatin components and association assays

Soluble chromatin (mean number of nucleosomes in a chain, *n = 6*) and core histones from chicken erythrocytes were isolated as described previously [3], [21]. Reconstituted mononucleosomes were assembled as described previously [3] using a salt gradient dialysis and Lowary and Widom ‘601’ DNA. Assembled mononucleosomes were stored on ice (4°C) and used within one week of assembly.

### Determination of kinetic parameters

The stoichiometry of inhibition (SI) and second-order association rate constants (*k*_ass_) were determined as described previously [2].

### Measurements of change in intrinsic tryptophan fluorescence

Potential co-factor-induced conformational change in MENT proteins was studied by monitoring the intrinsic fluorescence changes of the enzymes in the presence and absence of co-factor. MENT and variants (0.2 µM) were incubated with increasing amounts of the co-factor in 10 mM HEPES buffer pH 7.0, containing 0.5 mM EDTA, 40 mM NaCl, and 0.1% (w/v) Brij-35 in a final volume of 2 ml. Each experiment was repeated in triplicate. An excitation wavelength of 295 nm was used to minimise inner filter effects of measured fluorescence and DNA absorbance at excitation wavelengths was monitored and did not exceed 0.1 for any sample. The emission was scanned 5 times over the range of 300 to 400 nm at 60 nm/min, using excitation and emission slit widths of 5 nm. The emission spectrum of buffer in the presence and absence of cofactor, which was used as a control, was subtracted from the protein emission spectra. Acrylic cuvettes were used throughout to avoid adsorption to the cuvette walls.
